# Records of Maculated Typhus or Ship Fever, with Suggestions of Treatment

**Published:** 1852-12

**Authors:** J. B. Upham

**Affiliations:** Boston


*Records of Maculated Typhus or Ship Fever, with Suggestions of Treatment.* By J. B. Upham, M. D., of Boston. With Plates.

We have been remiss in not before acknowledging this *brochure*, reprinted from the New York Journal of Medicine, No. for July, 1852.

Dr. Upham, after a large experience, has arrived at the conclusion that *typhus* differs in essential points from *typhoid* fever. He calls attention to the occurrence of inflammation of the large intestine as an occasional complication, or sequel of typhus. The morbid appearances are represented by several finely lithographed plates.

The practical suggestions as to treatment appear to us to be judicious.

